# Lung Cancer Presenting as Acrometastasis to the Finger: A Case Report

**DOI:** 10.1155/2010/234289

**Published:** 2010-06-13

**Authors:** Lawrence Stephen Long, Leslea Brickner, Lisa Helfend, Tony Wong, Derek Kubota

**Affiliations:** ^1^Department of Medicine, Kaiser Permanente, Oakland, CA 94611, USA; ^2^Department of Pathology, Kaiser Permanente, Oakland, CA 94611, USA; ^3^Department of Radiology, Kaiser Permanente, Oakland, CA 94611, USA

## Abstract

Lung cancer is the commonest cause of acrometastatic disease to the fingers. Here we describe a case of occult lung cancer presenting as unrelenting finger pain and swelling from a metastatic phalangeal fracture. The patient's management was largely palliative and he died soon after discovery of the primary tumor. Digital acrometastatic disease rarely becomes symptomatic before the primary lung cancer is diagnosed and, as observed in this case, carries a very poor prognosis. Clinicians should be cognizant of the strong association between digital acrometastases and bronchogenic carcinoma and vigilant in screening high-risk patients with importunate finger symptoms.

## 1. Introduction

The bones of the finger rarely harbor metastatic disease, but when they do, it is a revealing clinical finding. Most bony metastases, including nondigital acrometastatic disease, arise from a wide array of primary tumors (e.g., prostate, lung, kidney, breast, gastrointestinal). By contrast, the etiology of digital acrometastases is almost exclusively bronchogenic carcinoma [1]. Fortunately, digital acrometastatic lung cancer is seldom seen, accounting for approximately one out of 500 lung cancers with bony metastases [2]. It carries a grim prognosis, with a mean survival of three to six months after presentation [3, 4].

Here we describe a case of occult lung cancer presenting as metastatic disease to the finger.

## 2. Case Presentation

**Figure 1:**
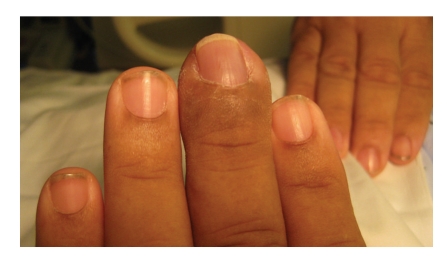
A swollen, painful tip of the left third finger.

A 53-year-old Guamanian-American man with seven pack-years of smoking, asbestos exposure as a child, and a family history of lung cancer suffered from a swollen, erythematous, painful tip of the left third finger. He first blamed the swelling on an embedded splinter, which he quickly dislodged with warm compresses and tweezers. However, the swelling persisted and the erythema deepened (see Figure 1). After two months of unremitting finger pain, he developed right hip pain subsequent to falling off a ladder. His primary care physician attributed the finger and hip pain to trauma, prescribed etodolac, and ordered a radiograph of the hip, which was unremarkable.

Two weeks later, the patient presented to the emergency department with sudden-onset chest pain, exacerbated by deep breathing. He was tachypneic, tachycardic, and slightly hypoxic. Computed tomography revealed a right upper lobe lung mass and right hilar adenopathy. Poorly differentiated adenosquamous carcinoma, characterized by positivity to mucin, CK7, TTF1, P63, and CK5/6, was seen on lung pathology.

**Figure 2:**
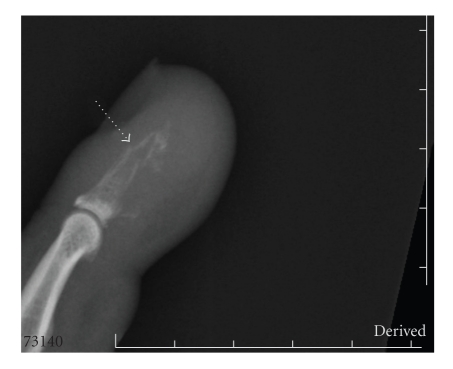
A pathologic fracture of the distal phalanx.

The patient received high-dose intravenous narcotics but nonetheless reported persistent finger pain. Orthopedics was consulted. Two radiographic views of the left hand showed a highly aggressive lytic lesion involving the distal phalanx with permeative margins and slight immature periosteal reaction. There was significant associated soft tissue swelling or mass, as well as an associated minimally displaced pathologic fracture through the midportion of the phalanx. Additionally, there were adjacent tiny ossific or calcific fragments, possibly displaced bony fragments or dystrophic soft tissue calcification (see Figure 2). Taken together, the radiographic findings argued strongly against a traumatic injury, but instead supported a destructive and infiltrating process, such as lytic metastasis. The patient declined biopsy, elected comfort measures only, and died one month later.

## 3. Discussion

Acrometastasis is reported infrequently, with only one out of 1000 bony metastases traveling to the hand [5]. The primary tumors most implicated, in order of prevalence, are lung, kidney, breast, and gastrointestinal [4]. Men are more likely to be affected than women, with solitary phalangeal lesions commonly observed [4]. 

The tumor cells are thought to migrate to the bones of the hand via blood, not lymphatics [6]. Although the wide hematogenous spread of many visceral organ tumors is often restricted by the hepatic and pulmonary capillary beds, malignant cells of the lung have unimpeded access to the distal arterial system. This may explain why the commonest source of acrometastic disease is bronchogentaic carcinoma [6].

While acrometastatic lung cancer is rare, occult lung cancer presenting as metastasis to the finger is even more exceptional. Although good epidemiologic data for lung cancer are unavailable, one small study found that approximately 10% of acrometastases became symptomatic before the primary tumor was identified [7].

The presentation of digital acrometastatic lesions varies. The affected finger may appear infected, with tenderness, erythema, heat, and swelling [8]. In addition, the overlying skin may weep, bleed, or ulcerate [3, 9]. Terminal phalanges of the dominant hand are most commonly involved [4, 10].

Importantly, bronchogenic metastases to bone are usually lytic in nature [4]. Although the current case did not have a biopsy-proven acrometastatic lesion, radiographic differential considerations were narrow and supported an aggressive lytic metastasis to bone in a patient with known lung cancer. Nonetheless, another process, such as acute osteomyelitis or primary neoplasm, could not be excluded without biopsy. 

As seen in this case, patients may report a history of trauma to the affected finger. Interestingly, some authors argue that a history of trauma is causal, not coincident, by introducing malignant cells to the bone through increased blood flow and the release of local chemotactic factors [11]. These factors, including prostaglandins, promote cell migration and adhesion to bone, and may create a conduit for metastatic disease.

Given the bleak prognosis of digital acrometastatic lung cancer, treatment is largely palliative. Amputation and chemotherapy have been used, but recent literature suggests that localized radiotherapy can successfully relieve pain and return function to the affected finger [4]. There may also be a targeted role for bone-remodeling pharmacotherapies, such as bisphosphonates or denosumab, which have demonstrated utility in treating other bony metastases [12, 13].

As this case illustrates, clinicians must be mindful in screening patients at risk for lung cancer with persistent digital symptoms.
